# Trends in the use of oral anticoagulants, antiplatelets and statins in four European countries: a population-based study

**DOI:** 10.1007/s00228-021-03250-6

**Published:** 2021-11-17

**Authors:** Luis A. García Rodríguez, Lucía Cea Soriano, Francisco J. de Abajo, Francesca Valent, Jesper Hallas, Miguel Gil, Chiara Cattaruzzi, Sara Rodriguez-Martin, Pareen Vora, Montse Soriano-Gabarró, David Gaist

**Affiliations:** 1 Spanish Centre for Pharmacoepidemiologic Research (CEIFE), Madrid, Spain; 2 Department of Public Health and Maternal and Child Health, Faculty of Medicine, Complutense University of Madrid), Madrid, Spain; 3 Clinical Pharmacology Unit, Department of Biomedical Sciences (Pharmacology Section), University Hospital Príncipe de Asturias, University of Alcalá (IRYCIS), Alcalá de Henares, Madrid, Spain; 4 Institute of Hygiene and Clinical Epidemiology, Udine Integrated University Health Unit, Udine, Italy; 5 Clinical Pharmacology and Pharmacy, Department of Public Health, University of Southern Denmark, Odense, Denmark; 6 División de Farmacoepidemiología Y Farmacovigilancia, Agencia Española de Medicamentos Y Productos Sanitarios, Madrid, Spain; 7 Pharmaceutical Service, Udine Integrated University Health Unit, Udine, Italy; 8 Bayer AG, Berlin, Germany; 9 Research Unit for Neurology, Odense University Hospital, University of Southern Denmark, Odense, Denmark

**Keywords:** Antithrombotics, Aspirin, Anticoagulants, Statins, Database, Trends

## Abstract

**Purpose:**

To evaluate time trends in the prevalence of antithrombotic and statin use in four European countries.

**Methods:**

Using population-based data from the United Kingdom, Denmark, Spain and Italy between 2010 and 2018, we calculated standardized annual prevalence proportions of antithrombotics and statin use, and changes in prevalence proportions (2018 vs. 2010).

**Results:**

Prevalence proportion of statins increased from 24.8% to 24.6% (UK), 21.0% to 22.3% (Region of Southern Denmark [RSD]), 12.9% to 14.3% (Udine, Italy), and 20.3% to 23.2% (Spain). Prevalence proportions of antithrombotics declined in all four countries: 18.7% to 15.9% (UK; − 2.8% points), 18.9% to 18.1% (RSD; − 0.8% points), 17.7% to 16.6% (Udine; − 1.1% points) and 15.0% to 13.6% (Spain; − 1.4% points). These declines were driven by reductions in low-dose aspirin use: 15.3% to 8.9% (UK; − 6.4% points), 16.3% to 9.5% (RSD; − 6.8% points), 13.5% to 11.6% (Udine; − 1.9% points), and 10.2% to 8.8% (Spain; − 1.4% points). In the UK, low-dose aspirin use declined from 9.1% to 4.3% (− 4.8% points) for primary CVD prevention, and from 49.6% to 36.9% (− 12.7% points) for secondary prevention. Oral anticoagulant use gradually increased but did not fully account for the decrease in low-dose aspirin use.

**Conclusions:**

Antithrombotic use in the UK, RSD, Udine and Spain declined between 2010 and 2018, driven by a reduction in use of low-dose aspirin that is not completely explained by a gradual increase in OAC use. Use of statins remained constant in the UK, and increased gradually in the RSD, Udine and Spain.

**Supplementary Information:**

The online version contains supplementary material available at 10.1007/s00228-021-03250-6.

## Introduction

Despite a decline in cardiovascular disease (CVD) mortality, CVD including ischaemic heart disease and stroke, remains the leading cause of death in Europe, accounting for approximately 45% of all deaths [1]. The burden of CVD – both direct and indirect costs – similarly remains high [1, 2]. While lifestyle changes, antithrombotic therapy, statins as lipid-lowering therapy, and antihypertensives remain the cornerstone of CVD prevention, the last decade has seen the introduction of the direct oral anticoagulants (DOACs) as a new class of oral anticoagulants (OACs) along with changes to CVD prevention guidelines. For example, the 2010 European Society of Cardiology (ESC) guidelines no longer recommended use of low-dose aspirin for thromboprophylaxis in patients with atrial fibrillation (AF) [3], while the recent 2019 ESC guidelines for the management of dyslipidaemia indicate the potential for statin use in broader patient populations [4]. Furthermore, while antiplatelet therapy with low-dose aspirin remains recommended for secondary prevention of CVD [5, 6], its use in primary CVD prevention is largely not recommended. European Society of Cardiology (ESC) guidelines continue to advise against prescribing low-dose aspirin for primary prevention [6, 7], yet some position statements have advocated a more nuanced approach and focus mainly on patients with high CVD and low-bleeding risks [8, 9].

Owing to these changes in CVD management strategies, it is important to monitor how these commonly used CVD drugs are being prescribed at the population-level; however, large population-based studies evaluating country-level trends in prescriptions rates are limited. In this study, we used population-based clinical data from four European countries to describe time-trends in the use of antithrombotics and statins over a contemporary 9-year period. Our main interest was in the use of antiplatelets (and particularly low-dose aspirin) as we hypothesised that we would observe a decline in their use over time due to the changes in guidelines. Anticoagulants and statins were selected for this study as examples of two other commonly used CVD therapies, in order to see whether similar or different trends were seen in their use as with antiplatelets.

## Methods

### Data sources

The study used patient-level data from four European countries that all have national healthcare systems – the United Kingdom (UK), Denmark, Italy and Spain – using longitudinal healthcare databases that are considered representative of the general population/region for that country: the IQVIA Medical Research Data (IMRD) database, linked registries covering the Region of Southern Denmark (RSD), the Friuli Venezia Giulia database of the Udine Integrated University Health Unit in Italy (FVG ASUIUD) and Base de Datos para la Investigación Farmacoepidemiológica en Atención Primaria (BIFAP) primary care database in Spain (see the Supplementary Method**s** for further details on the data sources). These data sources were chosen based on the principal investigator in each location being willing and having access to the data for the respective analyses.

### Patient and public involvement

There was no public or patient involvement in the design, conduct, reporting or dissemination plans of our research.

### Study population

The study population from each data source included individuals aged between 40 and 99 years of age for each calendar year within the 9-year study period from 1 January 2010 to 31 December 2018. This time period was chosen to enable analysis of the most recent available data from each data source while also providing a long enough period to identify any contemporary time trends. For the UK and Spanish analyses, only individuals with a permanent registration status with the primary care physician were included.

### Antithrombotics and statins

For each country-specific study population, we identified all prescriptions for antithrombotics (antiplatelets and OACs) and statins during the study period (see Supplementary Table 1 for codes). Antiplatelets were categorized as low-dose aspirin (75–325 mg tablets in the UK, 75–150 mg tablets in Denmark, 75–300 mg tablets in Italy, and 75 to 300 mg tablets in Spain), clopidogrel, or ‘other’ (ticagrelor, prasugrel, and triflusal). Oral anticoagulants included all vitamin K antagonists (VKAs) and DOACs available during the study period. To qualify as a user of one of these drug classes in a specific calendar year, individuals were required to have at least one prescription for the drug in that year.

### Statistical analysis

Annual prevalence proportions of use of each drug class were expressed as a percentage and were calculated by dividing the number of individuals with a prescription for the drug class in question in each calendar year by the number of individuals alive in the respective database on the 1 January of that calendar year. To enable valid comparisons between countries and between calendar years in each database, direct age- and sex-standardization was performed using the age (10-year age bands) and sex distribution from the Eurostat reference population [10]. For low-dose aspirin use, annual prevalence proportions were also stratified by age and sex. We calculated the percentage change – both absolute difference and relative difference – in prevalence proportion of each drug class over the study period. Relative differences in annual prevalence proportions was calculated by dividing the difference in prevalence between 2018 and 2010 by the prevalence in 2010 and multiplying by 100. In a post-hoc analysis of the data from the UK and Spain, we calculated annual prevalence proportions, and absolute percentage change in use (2018 vs. 2010) of low-dose aspirin according to whether it was prescribed for primary or secondary CVD prevention (see Supplementary Methods for details). We were unable to perform this post-hoc analysis for the RSD and Udine because we did not have data on the indication for low-dose aspirin use at our disposal in the datasets for these two countries. To do this, for each calendar year we considered individuals with a code for CVD any time before 1 January of that calendar year as having received low-dose aspirin for secondary CVD prevention; all remaining individuals were assumed to have received it for primary CVD prevention. Additionally, among the secondary CVD prevention population in the UK, we analysed prevalence rates by aspirin indication, e.g. stroke, myocardial infarction, ischaemic heart disease etc.) using the closest recorded CVD code before the first low-dose aspirin prescription in each calendar year to determine the indication. Analyses were undertaken using Stata version 12.0 (StataCorp. 2017).

## Results

### Statins and all antithrombotics

**Fig.1:**
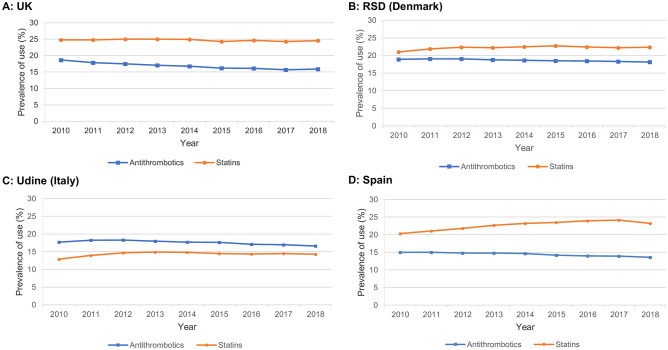
Annual prevalence proportion of antithrombotics and statins in **A** UK, **B** RSD (Denmark), **C** Udine (Italy), **D** Spain RSD, Region of Southern Denmark, UK, United Kingdom

**Fig. 2:**
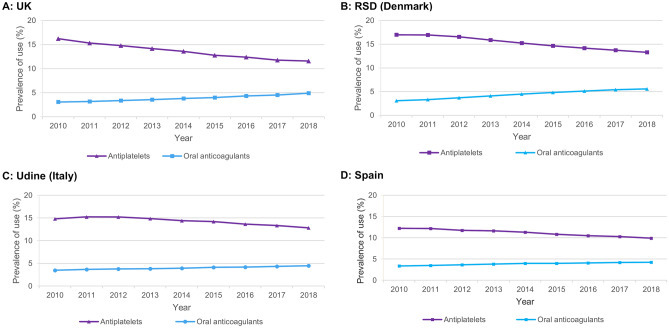
Annual prevalence proportion of antiplatelets and oral anticoagulants in **A** UK, **B** RSD (Denmark), **C** Udine (Italy), **D** Spain RSD, Region of Southern Denmark, UK, United Kingdom

Over the study period, the use of statins remained broadly consistent in the UK (24.8% in 2010 and 24.6% in 2018, dipping slightly from 2015–2018) and increased slightly in the RSD (21.0% to 22.3%), Udine (12.9% to 14.3%) and Spain (20.3% to 23.2%) (Fig. 1). Statin use was consistently higher than antithrombotic use in the UK, RSD and Spain, and consistently lower in Udine. A trend over time was seen in a reduction in use of antithrombotics in all four countries, but most notably in the UK, dropping to a lower level than the other three countries by 2018: decreases were 18.7% to 15.9% (UK; − 2.8% points), 18.9% to 18.1% (RSD; − 0.8% points), 17.7% to 16.6% (Udine; -1.1% points) and 15.0% to 13.6% (Spain; − 1.4% points) (Fig. 1). The declines in antithrombotic use across the study period were driven by a decrease in use of antiplatelets; decreases were 16.2% to 11.6% (UK; − 4.6% points), 17.0% to 13.3% (RSD; − 3.7% points), 14.8% to 12.8% (Udine; − 2.0% points) and 12.2% to 9.9% (Spain; − 2.3% points) (Fig. 2). In contrast, OAC use steadily increased in all four countries: 3.1% to 4.9% (UK; + 1.8% points), 3.1% to 5.6% (RSD; + 2.5% points), 3.5% to 4.5% (Udine; + 1.0% points) and 3.4% to 4.2% (Spain; + 0.8% points) (Fig. 2). The relative change (2018 vs. 2010) in prevalence proportions antiplatelets, OACs, and statins is shown in Supplementary Fig. 1 and Supplementary Table 2; the prevalence proportion of each class of drugs in the last year of the study period (2018) is shown in Supplementary Fig. 2.

### Oral anticoagulants

**Fig. 3:**
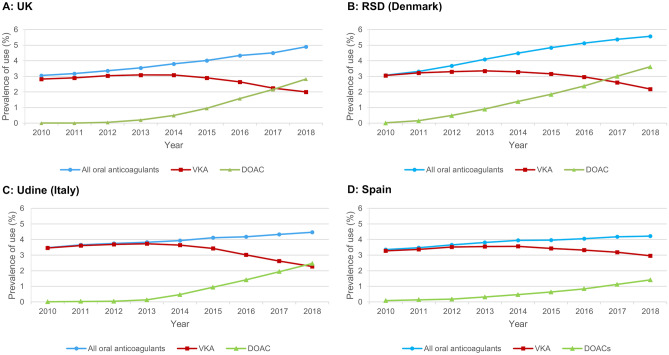
Annual prevalence proportion of oral anticoagulants in **A** UK, **B** RSD (Denmark), **C** Udine (Italy), **D** Spain RSD, Region of Southern Denmark, UK, United Kingdom

The increases in OAC use across the study period were driven by an increased uptake of DOACs, corresponding with decreases in VKA use (Fig. 3); use of DOACs had overtaken VKAs by 2017 in the UK and RSD, and by 2018 in Udine. VKAs remained the main OAC of use in Spain across study years.

### Antiplatelets

**Fig. 4:**
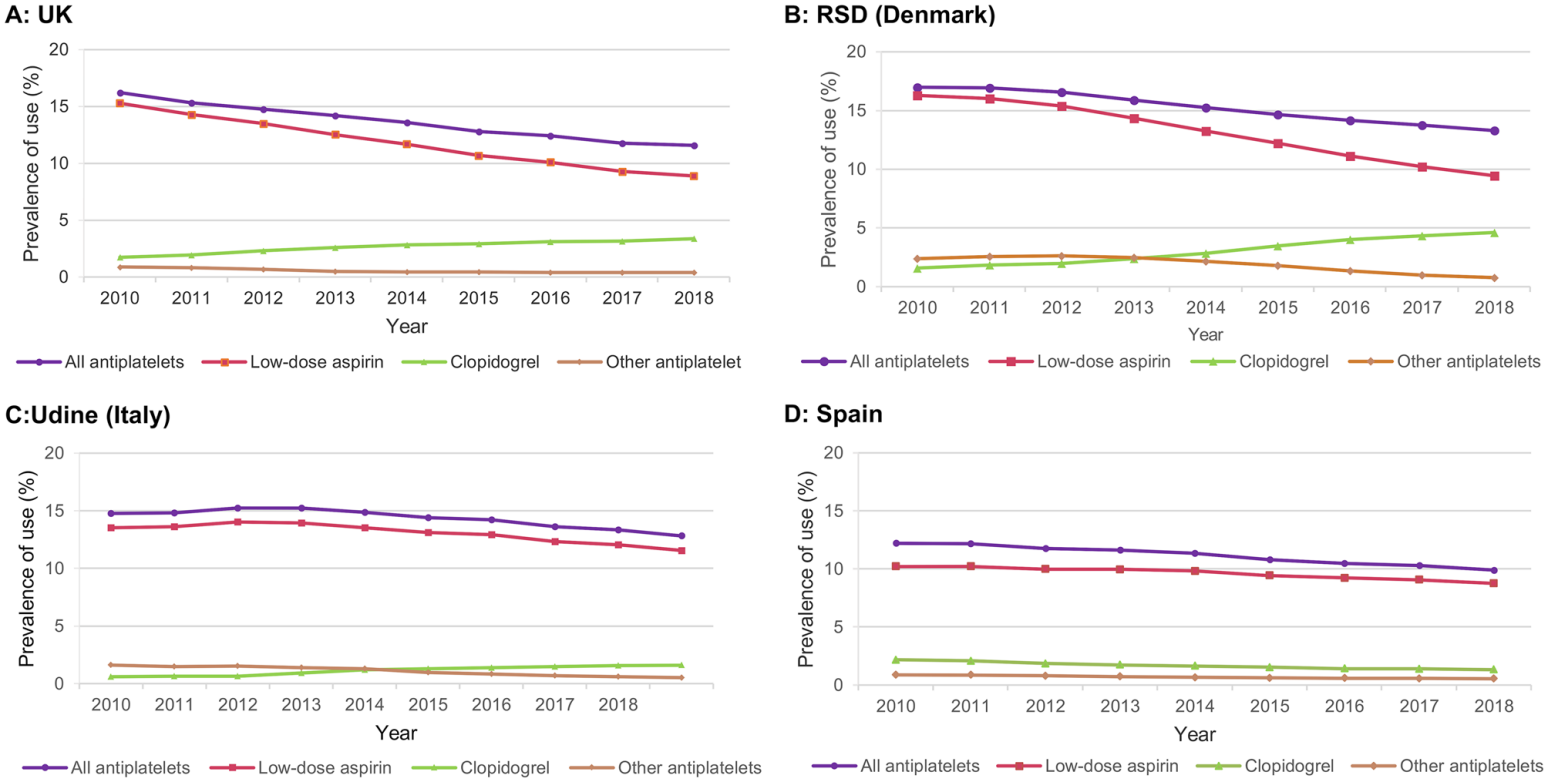
Annual prevalence proportion of antiplatelets in **A** UK, **B** RSD (Denmark), **C** Udine (Italy), **D** Spain RSD, Region of Southern Denmark, UK, United Kingdom

The observed declines in antiplatelet use were driven by a decrease in low-dose aspirin use: 15.3% to 8.9% (UK; − 6.4% points), 16.3% to 9.5% (RSD; − 6.8% points), 13.5% to 11.6% (Udine; − 1.9% points), and 10.2% to 8.8% (Spain; − 1.4% points), and this was apparent in both males and females **(**Supplementary Fig. 3). In the UK and RSD, greater reductions in low-dose aspirin use were seen with increasing age; however, age group differences were minimal in Udine and Spain (**S**upplementary Fig. 4). Use of clopidogrel increased in the UK (1.7% to 3.4%; + 1.7% points), RSD (1.6% to 4.6%; + 3.0% points) and Udine (0.6% to 1.6%; + 1.0% points), and decreased slightly in Spain (2.2% to 1.3%; − 0.9% points) (Fig. 4). The relative change (2018 vs. 2010) in the prevalence proportion of different antiplatelets is shown in Supplementary Fig. 5.

#### Primary and secondary CVD prevention

In the post-hoc analysis, using data from the UK and Spain, low-dose aspirin use for primary CVD prevention declined from 9.1% to 4.3% (− 4.8% points) in the UK and from 6.7% to 5.5% (− 1.2% points) in Spain (Supplementary Fig. 6). Use of low-dose aspirin for secondary CVD prevention declined in the UK (49.6% to 36.9%; − 12.7% points) and increased very slightly in Spain (46.6% to 47.1%; + 0.5% points). **(**Supplementary Fig. 7). Use of clopidogrel for secondary CVD prevention use increased in the UK (8.7% to 17.5%; + 8.8% points) and decreased in Spain (17.8% to 10.0%; − 7.8% points) (Supplementary Fig. 7).The decline in use of low-dose aspirin for secondary CVD prevention in the UK was driven by a reduction in prescribing to patients with a record of stroke (65.1% points to 24.4% points; − 40.7% points); for patients with a record of MI/unstable angina/coronary revascularisation, prevalence of low-dose aspirin use declined from 77.5% to 69.0% (− 8.5% points).The relative change (2018 vs. 2010) in the prevalence proportion of low-dose aspirin for primary CVD prevention was − 53.5% in the UK and − 20.1% in Spain; for secondary CVD prevention it was − 25.6% in the UK and + 1.1% in Spain.

## Discussion

In this population-based study across different European healthcare systems we observed a notable decline in the use of antithrombotics between 2010 and 2018 in all four countries, and while a small steady increase in statin use was seen in the RSD, Udine and Spain, use of statins remained broadly consistent in the UK. The observed declines in antithrombotic use were driven by decreasing use of low-dose aspirin, and in the UK and RSD, this was clearly driven by a reduction in use among the elderly, suggesting possible patient/physician concerns about bleeding. Although each country saw a gradual steady increase in OAC use over the study period, this did not completely account for the level of decline in low-dose aspirin use; for example, absolute reductions in low-dose aspirin prevalence of 6.4% (UK) and 6.8% (RSD) corresponded with increases of 1.8% (UK) and 2.5% (RSD) in OAC use.

We are aware of few other studies on this topic; however, two recent studies have similarly described a temporal trend of decreasing low-dose aspirin prevalence. In Wales, Protty et al. [11] found that low-dose aspirin prescription rates decreased by 15% from 2005–2016. In a study based on US National Health Interview Survey data from the United States, self-reported aspirin use dropped from 32.6% in 2015 to 30.0% in 2012, with a slighter larger drop seen for primary CVD prevention [12]. The decline in low-dose aspirin use for primary CVD prevention seen in our study for the UK and Spain, could be partly related to the 2009 publication of the Antithrombotic Trialists’ Collaboration meta-analysis [13], which showed an uncertain absolute net value of low-dose aspirin in primary prevention due to higher bleeding risks [13]. Another possible explanation is the ESC’s steadfast recommendation to avoid low-dose aspirin in individuals without prevalent CVD [6, 7, 14], along with changes in the 2010 ESC guidelines relating to low-dose aspirin use in AF [3], as shown in previous studies [15, 16]. Although one could speculate that better control of CVD risk factors might explain declining low-dose aspirin use, further investigation would be needed in this area because reductions in some risk factors, such as hypertension and smoking, in high-income European countries have been accompanied by increases in others, for example, in diabetes and obesity [2]. The clear decline in low-dose aspirin use for secondary CVD prevention in the UK was not seen in Spain, which instead saw a reduction in clopidogrel use. In our analysis of UK secondary prevention data, the largest reduction in low-dose aspirin prescribing was to patients with stroke, with minor reductions to patients with other indications such as myocardial infarction. This reduction of low-dose aspirin use in stroke patients was partially compensated with an increase in clopidogrel and OAC use.

Statins are well-established as effective in the primary and secondary prevention of CVD [17–20], and have been consistently recommended in ESC guidelines for high-risk patients [4, 7]. By 2018, prevalence of statin use was similar in the UK, RSD and Spain, being prescribed to between a fifth and a quarter of the population, compared with 1 in 7 individuals in Udine. The gradual small increase in statin use observed in the RSD, Udine and Spain, was not seen in the UK, albeit use remained the highest in the UK across study years. We are unaware of other studies describing contemporary trends in statin prescribing in Europe, with previous studies on the topic set in earlier time periods [21, 22]. The steady increases in OAC use for AF/VTE indications seen in our study have similarly been observed in other studies in Europe [15, 23, 24]. These increases were driven by a greater uptake of DOACs, in line with several previous reports from Europe [15, 16, 23–26] and America [27–29], indicating increasing confidence in the use of DOACs over VKAs. By 2018, levels of OAC use were broadly similar between countries in our study, ranging between 4.2% in Spain to 5.6% in the RSD. Although our study did not aim to compare drug use between individual countries, but rather describe trends within each country, it is still noteworthy to mention that several other factors, aside from guidelines and publication of pivotal studies may have impacted prescribing, for example, budgeting, reimbursement restrictions, introduction of generics, and different prescribing habits of physicians.

To our knowledge, our study, based on data from 2010–2018, is the first to make direct comparisons in the contemporary use of antithrombotics and statins between European countries with different healthcare structures. We used large population-based samples representative of the respective wider national/regional population, and applied age- and sex-standardisation to enable valid inter-country and inter-year comparisons, accounting for any effects from differences/changes in the demographic structures. It is important to note, however, that the findings from Italy may not be representative of regions outside Friuli Venezia Giulia, as indicated by a recent report from the Italian Medicines Agency that shows large inter-region variability in the use of some cardiovascular drugs [30]. Another small limitation is that drug use during hospitalisations will not have been captured in the databases; however, antithrombotics and statins are long-term therapies prescribed in primary care and therefore misclassification levels will have been minimal. Also, we did not have data to our disposal to evaluate primary/secondary CVD prevention in the RSD and Udine, and therefore were unable to evaluate whether they followed the respective prescribing trends seen in the UK or Spain.

In conclusion, antithrombotic use in the UK, RSD, Udine and Spain has declined between 2010 and 2018, driven by a reduction in use of low-dose aspirin that is not completely explained by a gradual increase in OAC use. This reduction in low-dose aspirin use was much more pronounced for patients without CVD, and because these patients accounted for the majority of patients analysed, the overall trend largely reflects the changes among the primary CVD prevention population. At the same time, use of statins has remained rather constant, increasing to a small extent in the RSD, Udine and Spain. Further studies of the prevalence of low-dose aspirin use for primary/secondary CVD prevention would help explore this topic further. Analyses of the wider-scale uptake of lifestyle changes for CVD prevention, and scale of bleeding concerns around preventative aspirin use, may also help to understand our study’s findings.

## Supplementary Information

Below is the link to the electronic supplementary material.

Supplementary file1 (PDF 234 KB)

Supplementary file2 (PDF 113 KB)

## Data Availability

Data are available from the corresponding author upon reasonable request.

## Code availability

Code is available from the corresponding author upon reasonable request.
