# Supplementary material for: Trends in the use of oral anticoagulants, antiplatelets and statins in four European countries: a population-based study
Source: Eur J Clin Pharmacol. 2021 Nov 17;78(3):497–504. doi: 10.1007/s00228-021-03250-6 (PMC8818635; doi:10.1007/s00228-021-03250-6)
Supplement: Supplementary file 1 — Supplementary file1 (PDF 234 KB) [file 228_2021_3250_MOESM1_ESM.pdf]

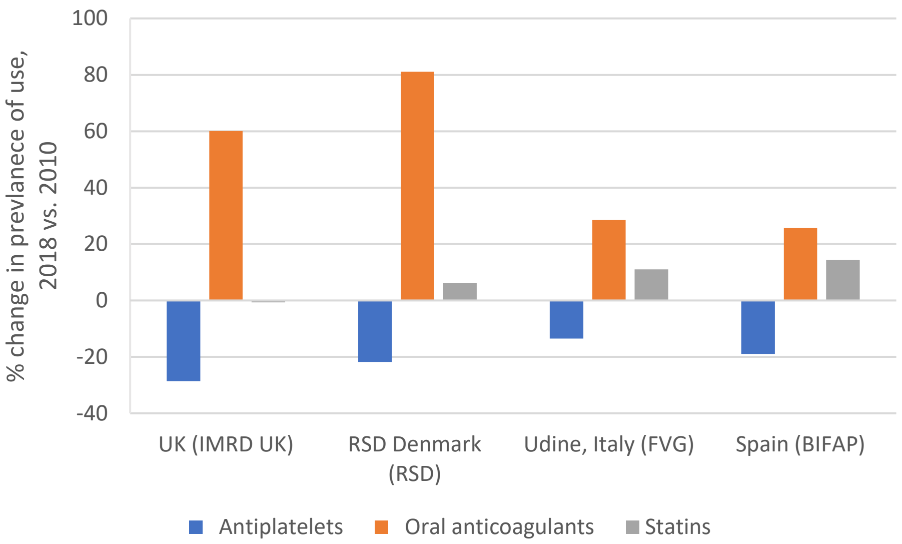

Supplementary Figure 1. Relative percentage change (2018 vs. 2010) in prevalence proportion of antiplatelets, oral anticoagulants, and statins in the UK, RSD (Denmark), Udine (Italy) and Spain.

Note: The relative difference in prevalence proportion for statins in the UK was -0.7%, and hence is not visible on the graph.

BIFAP, Base de Datos para la Investigación Farmacoepidemiológica en Atención Primaria; FVG, Friuli Venezia Giulia; RSD, Region of Southern Denmark, UK, United Kingdom

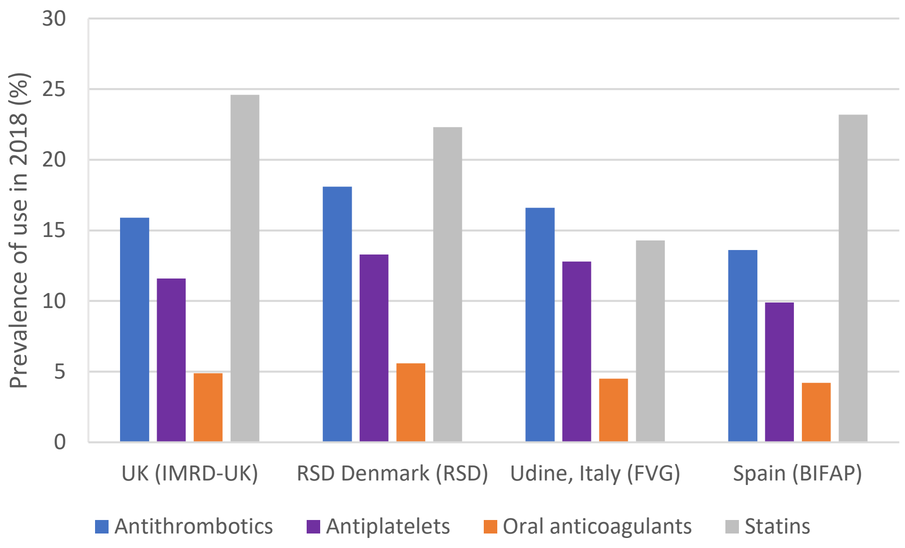

Supplementary Figure 2. Prevalence proportion of antithrombotics, antiplatelets, oral anticoagulants and statins in 2018 by country.
BIFAP, Base de Datos para la Investigación Farmacoepidemiológica en Atención Primaria; FVG, Friuli Venezia Giulia; RSD, Region of Southern Denmark, UK, United Kingdom

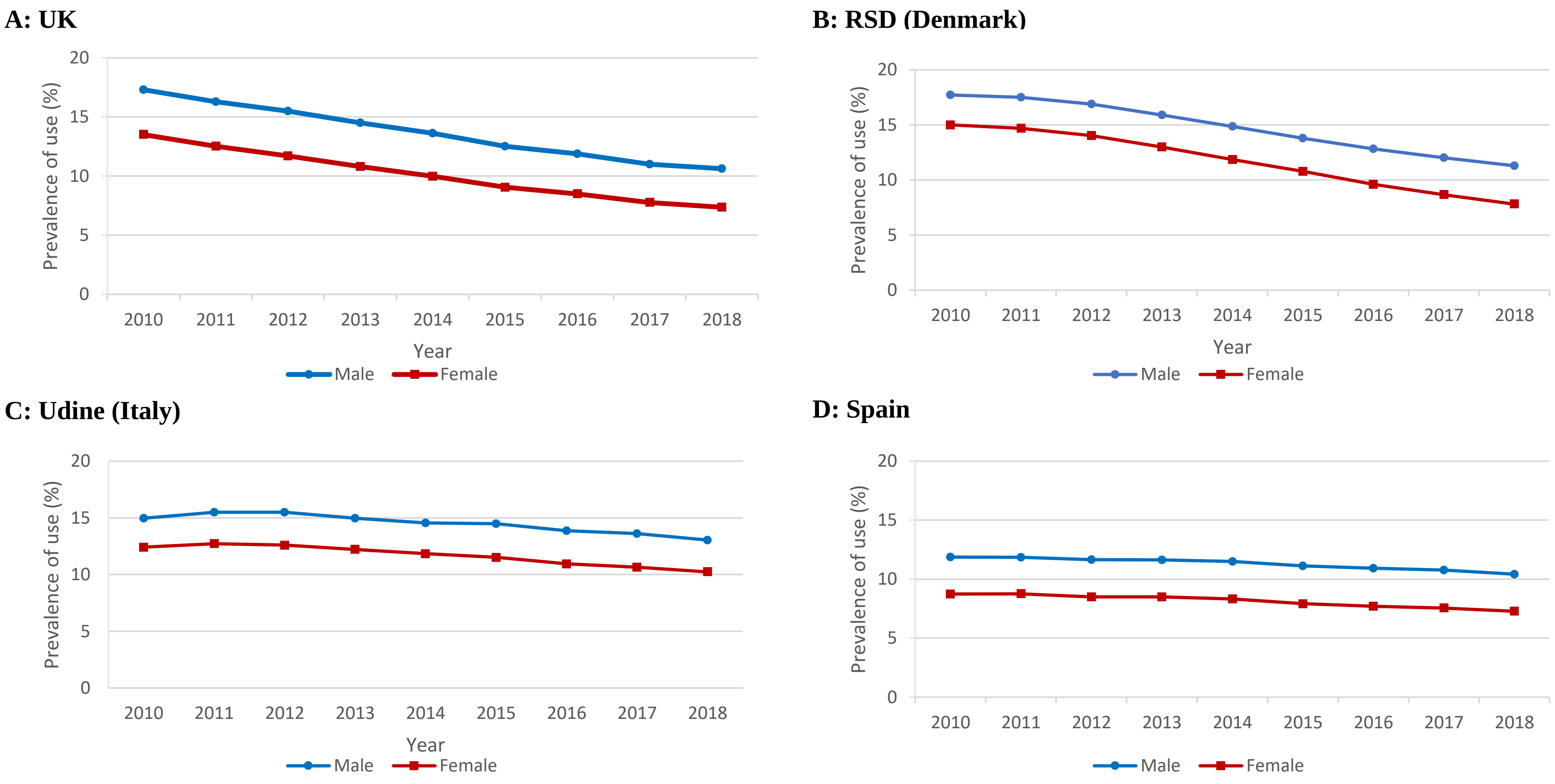

Supplementary Figure 3. Annual prevalence proportion of low-dose aspirin by sex in **(A)** the UK, **(B)** RSD (Denmark), **(C)** Udine (Italy), **(D)** Spain.

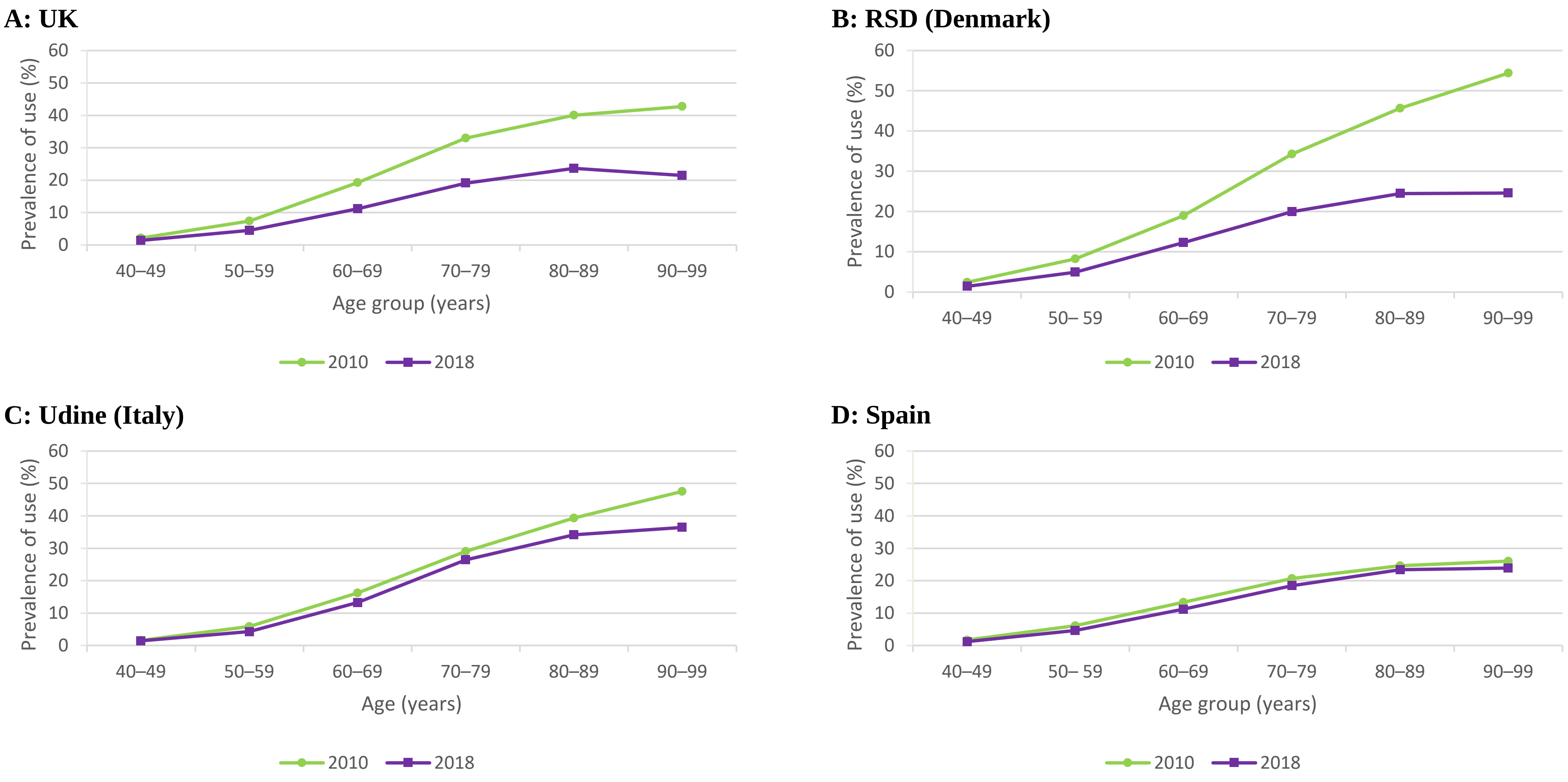

Supplementary Figure 4. Annual prevalence proportion of low-dose aspirin in 2010 and 2018 by age in **(A)** UK, **(B)** RSD (Denmark), **(C)** Udine, Italy**,** **(D)** Spain.

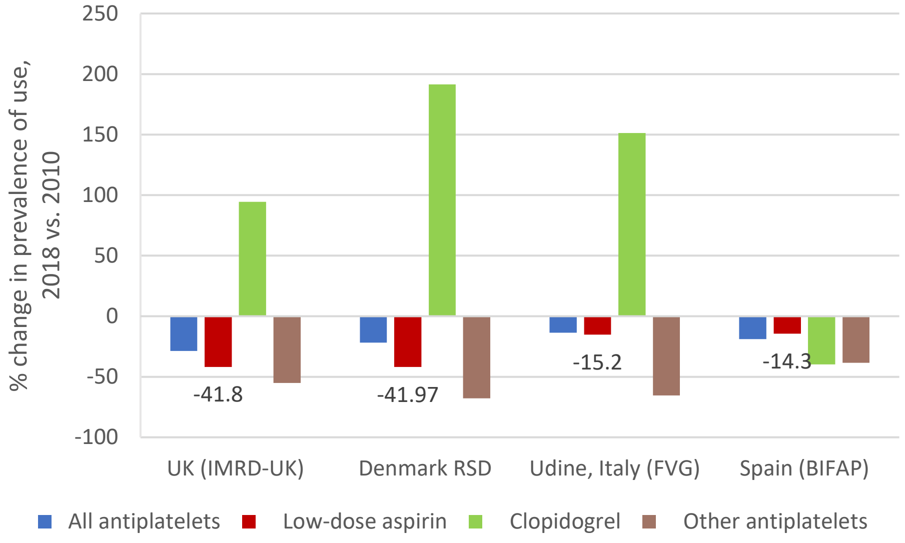

Supplementary Figure 5. Relative percentage change (2018 vs. 2010) in prevalence proportion of antiplatelet types in the UK, RSD (Denmark), Udine (Italy) and Spain.

BIFAP, Base de Datos para la Investigación Farmacoepidemiológica en Atención Primaria; FVG, FVG, Friuli Venezia Giulia; RSD, Region of Southern Denmark, UK, United Kingdom

A: UK

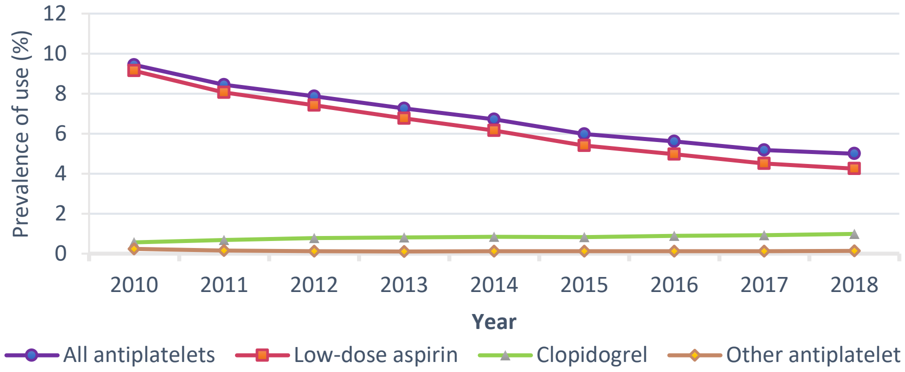

B: Spain

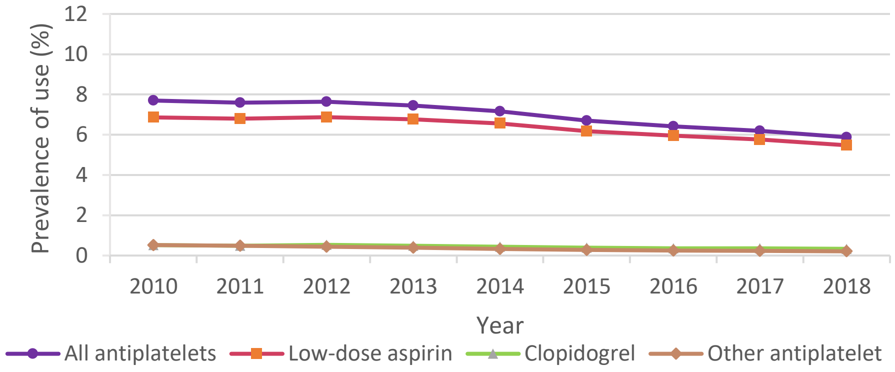

Supplementary Figure 6. Annual prevalence proportion of antiplatelet use for primary CVD prevention in (**A**) the UK, (**B**) Spain.

A: UK **B: Spain**

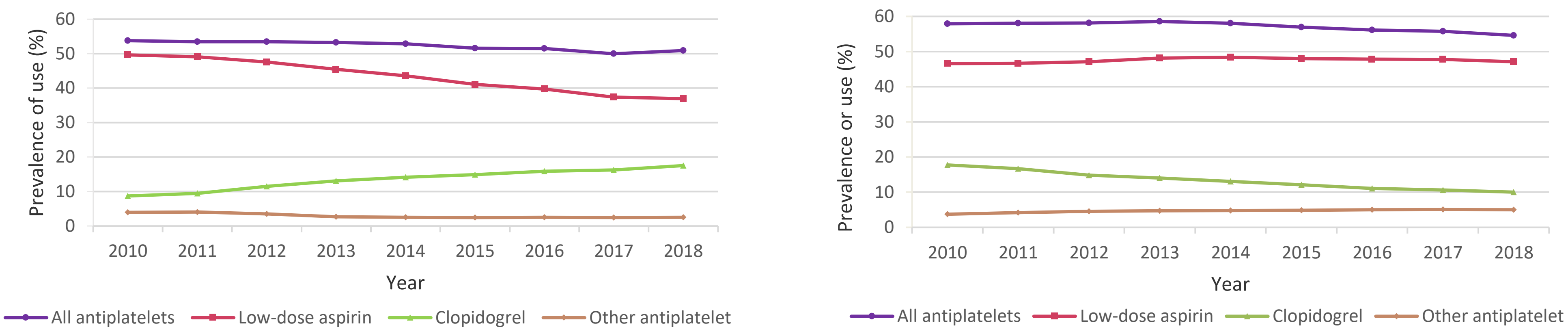

Supplementary Figure 7. Annual prevalence proportion of antiplatelet use for secondary CVD prevention in (**A**) the UK, (**B**) Spain.

Supplementary Table 1. Codes used to identify prescription for antiplatelets, anticoagulants and statins.

MEDICATION	ATC CODE
**Antiplatelets**	
ditazole	B01AC01
cloricromen	B01AC02
picotamide	B01AC03
clopidogrel	B01AC04
ticlopidine	B01AC05
acetylsalicylic acid	B01AC06
dipyridamole	B01AC07
carbasalate calcium	B01AC08
epoprostenol	B01AC09
indobufen	B01AC10
iloprost	B01AC11
abciximab	B01AC13
aloxiprin	B01AC15
eptifibatide	B01AC16
tirofiban	B01AC17
triflusal	B01AC18
beraprost	B01AC19
treprostinil	B01AC21
prasugrel	B01AC22
cilostazol	B01AC23
ticagrelor	B01AC24
cangrelor	B01AC25
vorapaxar	B01AC26
selexipag	B01AC27
combinations	B01AC30
acetylsalicylic acid, combinations with proton pump inhibitors	B01AC56
**Anticoagulants**	
Vitamin K antagonists	B01AA
dicoumarol	B01AA01
phenindione	B01AA02
warfarin	B01AA03
phenprocoumon	B01AA04
acenocoumarol	B01AA07
ethyl biscoumacetate	B01AA08
clorindione	B01AA09
diphenadione	B01AA10
tioclomarol	B01AA11
fluindione	B01AA12
**Direct thrombin inhibitor**	B01AE
dabigatran etexilate	B01AE07
**Direct factor Xa inhibitors**	B01AF
rivaroxaban	B01AF01
apixaban	B01AF02

MEDICATION	ATC CODE
edoxaban	B01AF03
**Statins**	
Statins	C10AA
Statins in combination with other lipid-lowering drugs	C10BA

Supplementary Table 2. Relative percentage change over the study period (2018 vs. 2010) in the prevalence proportion of antithrombotics and statin use in the UK, RSD (Denmark), Udine (Italy) and Spain.

	**UK**	**RSD (Denmark)**	**Udine (Italy)**	**Spain**
**All antithrombotics**	**-14.9**	**-4.2**	**-6.0**	**-9.3**
**Antiplatelets**	**-28.6**	**-21.76**	**-13.5**	**-18.9**
Low-dose aspirin	-41.8	-41.97	-15.2	-14.3
Clopidogrel	94.6	191.5	151.3	-39.7
Other antiplatelets*	-55.1	-67.9	-65.4	-38.5
**Oral anticoagulants**	**60.1**	**81.1**	**28.5**	**25.7**
DOACs	38091.9	17466.0	29203.3	1642.2
VKAs	-29.1	-28.4	-34.2	-9.7
**Statins**	**-0.7**	**6.3**	**11.0**	**14.4**

CVD, cardiovascular disease; DOAC, direct oral anticoagulant; OAC, oral anticoagulant; RSD, Region of Southern Denmark; UK, United Kingdom; VKA, vitamin K antagonist
