# Supplementary material for: Trends in the use of oral anticoagulants, antiplatelets and statins in four European countries: a population-based study
Source: Eur J Clin Pharmacol. 2021 Nov 17;78(3):497–504. doi: 10.1007/s00228-021-03250-6 (PMC8818635; doi:10.1007/s00228-021-03250-6)
Supplement: Supplementary file 2 — Supplementary file2 (PDF 113 KB) [file 228_2021_3250_MOESM2_ESM.pdf]

SUPPLEMENTARY METHODS

Data sources

United Kingdom: the IQVIA Medical Research Database (IMRD; approximately 3 million inhabitants)

The IMRD incorporates data from THIN (A Cegedim Database) which is an anonymised database of longitudinal primary care electronic health records (EHRs) derived from approximately 500 contributing general practices across the UK. As of September 2017, the database contains the pseudonymised primary care records of over 17 million patients in the UK, 3.1 million of which are actively registered in a contributing GP practice.(1) This corresponds to approximately 6% of the UK population.(2) The database is generalizable to the UK population.(3)The IMRD contains coded entries (Read codes, the standard clinical coding system used by the UK's National Health Service),(4) and captures all prescriptions issued in primary care. Information from secondary care visits is communicated to the general practitioner and entered in the patient's primary care record retrospectively.

Denmark: Registries from the Region of Southern Denmark (RSD; 1.2 million inhabitants)

Several Danish nationwide registries are available that cover the Region of Southern Denmark (RSD) – a population of approximately 1.2 million, and which is representative of the Danish population regarding demographic characteristics, healthcare utilization, and medication use.(5) This study used information from two registries – The Danish Civil Registration Service and the Danish National Prescription Registry. The Danish Civil Registration Service contains information on residency history, migration and vital status beginning in the 1960s.(6) The Danish National Prescription Registry contains information of prescriptions dispensed from community pharmacies since 1995, including the anatomical therapeutic code (ATC),(7) although the indication and prescribed dose are not available. The registries can be linked at the individual level by residents' unique personal identification number.

Italy: Friuli Venezia Giulia database of the Local Health Agency of Udine in Italy (FVG-ASUIUD; 250,000 inhabitants)

The Friuli Venezia Giulia database of the Local Health Agency of Udine in Italy (FVG-ASUIUD holds information from several administrative health databases covering the North-Eastern region of Friuli-Venezia-Giulia, which can be linked at an individual level through an encrypted unique identifier. The administrative databases cover both primary and secondary care. The FVG database is a well-established data source that has been used for pharmacoepidemiological research in several studies.(8–12)] Three databases will be used in this study: the Hospital Service Database, the Outpatient Prescription Database, and the Patient Identification Database. The Hospital Service Database contains data from all National Health System affiliated public and private hospitals within the region since 1986. The data include vital status at discharge, one primary diagnosis, up to five secondary diagnoses, and the dates of admission and discharge. The Outpatient Prescription Database contains data of reimbursed prescription medication dispensed from pharmacies in the region since 1995. The

Patients Identification Database contains demographic and vital status information of the region's residents from 1979.

Spain: BIFAP database

BIFAP (Base de Datos para la Investigación Farmacoepidemiológica en Atencion Primaria) is a longitudinal population-based database of computer-based medical patient records, from primary care physicians (PCP) belonging to the National Health System), and situated in any one of the 10 participating Autonomous Communities (Regions) throughout Spain (www.bifap.org).(13) BIFAP includes information routinely collected by PCP's in their practices (real world data). In 2018, the BIFAP database included anonymized and prospectively recorded data in the electronic medical records (EMR) of 7566 PCPs (6419 general practitioners (GP)/1,147 pediatricians) up to the end of 2018. The valid study period in BIFAP starts in 2001 when the EMRs where fully implemented throughout Spain. Information before EMR implementation is available as registered by the GP.

The BIFAP database is updated yearly. The total number of patients available for studies in the 2018 database is 12 million (2.3 million pediatric patients). The mean follow-up of patients in the database is 8.6 years and, the number of patients with follow-ups of 5 years or longer, is 8.9 million. The number of patients with up-to-date information in 2018 (active patients) is 8 million, representing 17 % of the total Spanish population. This percentage increases to 57.6 % if only the seven regions currently providing EMR data are considered. The whole population of five Spanish regions' is currently covered (population-based scheme) and inclusion of the whole population of the other participants' regions is foreseen within the next two years.

The PCPs' electronic medical records contain details on demographics, prescriptions, episodes of care (diagnoses/symptoms), specialist referrals, lifestyle information, date of death, other additional health data (test results, interventions, etc.) and clinical notes registered as free text. Diagnoses are classified according to the International Classification of Primary Care (ICPC)-2 and ICD-9 code system, depending on the participant region EMR software. Prescriptions are coded according to the Anatomical Therapeutic Chemical (ATC) classification system and linked to a diagnosis. Electronic dispensings are available for more than 75% of prescriptions through the linkage with community pharmacies registries.

BIFAP is in the process of linking primary care records with records from other data sources, specifically coded diagnoses at hospital discharge. To date, hospitalizations are available for a subset of periods and regions participating in BIFAP, representing around half of the BIFAP population in the five most recent years. Nevertheless, hospital diagnoses are likely to be registered in primary healthcare records, given the role of gatekeeper of the Primary Care Physicians in the context of the Spanish NHS.(13)

Identification of indication for low-dose aspirin use (primary/secondary cardiovascular disease prevention) in the post-hoc analysis.

For the datasets from the UK (the IQVIA Medical Research Data database) and Spain (BIFAP database), individuals with a coded entry for myocardial infarction, unstable angina, revascularization procedures, cerebrovascular disease, peripheral artery disease and ischaemic heart disease unspecified) any time before 1 January of the respective calendar were considered as having cardiovascular disease (CVD), and those with a prescription for low-dose aspirin in that calendar year were considered as taking it for secondary CVD prevention. All other individuals with a prescription for low-dose aspirin in the respective calendar year were considered as taking it for primary CVD prevention.

References

1. Health Research Authority. IQVIA Medical Research Data. https://www.hra.nhs.uk/planning-and-improving-research/application-summaries/research-summaries/the-health-improvement-network-thin-database/.
2. THIN. The Health Improvement Network (THIN). https://www.the-health-improvement-network.com/.
3. Blak BT, Thompson M, Dattani H, Bourke A. Generalisability of The Health Improvement Network (THIN) database: demographics, chronic disease prevalence and mortality rates. Inform Prim Care. 2011;19(4):251–5.
4. NHS Digital. Read codes.:http://systems.digital.nhs.uk/data/uktc/readcodes.
5. Henriksen DP, Rasmussen L, Hansen MR, Hallas J, Pottegård A. Comparison of the Five Danish Regions Regarding Demographic Characteristics, Healthcare Utilization, and Medication Use—A Descriptive Cross-Sectional Study. PLOS ONE. 2015;10(10):e0140197.
6. Pedersen CB. The Danish Civil Registration System. Scand J Public Health. 2011 Jul;39(7 Suppl):22–5.
7. Pottegård A, Schmidt SAJ, Wallach-Kildemoes H, Sørensen HT, Hallas J, Schmidt M. Data Resource Profile: The Danish National Prescription Registry. Int J Epidemiol. 2017 Jun 1;46(3):798-f.
8. Palese F, Pisa FE. Validation of discharge diagnosis coding for amyotrophic lateral sclerosis in an Italian regional healthcare database. Amyotroph Lateral Scler Frontotemporal Degener. 2020 Apr 22:1–7.
9. Birri S, Bidoli E, Zucchetto A, Dal Maso L, Zanier L, Serraino D. I TUMORI IN FRIULI VENEZIA GIULIA Dati di incidenza, sopravvivenza e prevalenza: aggiornamento al 2007. CANCER IN FRIULI VENEZIA GIULIA Incidence, survival, and prevalence data: updates as of 2007 2011.
10. Valent F, Tullio A, Errichetti E, Stinco G. The epidemiology of psoriasis in an Italian area: population-based analysis of administrative data. G Ital Dermatol Venereol. 2018 Jun 29.
11. Valent F. New oral anticoagulant prescription rate and risk of bleeding in an Italian region. Pharmacoepidemiol Drug Saf. 2017 Oct;26(10):1205–12.
12. Valent F. Diabetes mellitus and cancer of the digestive organs: An Italian population-based cohort study. J Diabetes Complications. 2015 Nov-Dec;29(8):1056–61.
13. Maciá-Martínez M-A, Gil M, Huerta C, Martín-Merino E, Álvarez A, Bryant V, et al. Base de Datos para la Investigación Farmacoepidemiológica en Atención

Primaria (BIFAP): A data resource for pharmacoepidemiology in Spain. Pharmacoepidemiology and Drug Safety. 2020;n/a(n/a):1–10.
